# Annotation of phenotypic diversity: decoupling data curation and ontology curation using Phenex

**DOI:** 10.1186/2041-1480-5-45

**Published:** 2014-11-05

**Authors:** James P Balhoff, Wasila M Dahdul, T Alexander Dececchi, Hilmar Lapp, Paula M Mabee, Todd J Vision

**Affiliations:** National Evolutionary Synthesis Center, Durham, NC USA; Department of Biology, University of North Carolina, Chapel Hill, NC USA; Department of Biology, University of South Dakota, Vermillion, SD USA

**Keywords:** Annotation, Phenotypes, Ontology, Curation, Systematics, Character matrix

## Abstract

**Background:**

Phenex (http://phenex.phenoscape.org/) is a desktop application for semantically annotating the phenotypic character matrix datasets common in evolutionary biology. Since its initial publication, we have added new features that address several major bottlenecks in the efficiency of the phenotype curation process: allowing curators during the data curation phase to provisionally request terms that are not yet available from a relevant ontology; supporting quality control against annotation guidelines to reduce later manual review and revision; and enabling the sharing of files for collaboration among curators.

**Results:**

We decoupled data annotation from ontology development by creating an Ontology Request Broker (ORB) within Phenex. Curators can use the ORB to request a provisional term for use in data annotation; the provisional term can be automatically replaced with a permanent identifier once the term is added to an ontology. We added a set of annotation consistency checks to prevent common curation errors, reducing the need for later correction. We facilitated collaborative editing by improving the reliability of Phenex when used with online folder sharing services, via file change monitoring and continual autosave.

**Conclusions:**

With the addition of these new features, and in particular the Ontology Request Broker, Phenex users have been able to focus more effectively on data annotation. Phenoscape curators using Phenex have reported a smoother annotation workflow, with much reduced interruptions from ontology maintenance and file management issues.

## Background

Phenex [1] is a desktop application for creating semantic annotations within the phenotypic character matrix datasets common in evolutionary biology. Using terms from a user-configurable set of ontologies, free-text character state descriptions can be annotated using the Entity–Quality methodology for ontologically describing phenotypes [2, 3]. In addition, taxon entries can be annotated with identifiers from a taxonomy ontology [4]. Phenex exploits the ability of the NeXML file format [5] to attach arbitrary metadata, including ontological expressions, to phylogenetic data elements, to embed these ontology annotations within traditional character matrix data. Phenex was developed as part of the Phenoscape project [6], and it has been used to connect the morphological diversity of vertebrates to model organism phenotypes via common ontological semantics [7, 8].

Since Phenex was initially described, we have added several substantial new features that collectively aim to address major bottlenecks in the efficiency of the phenotype curation workflow. Specifically, three main issues led to the development of the following three features:

1. Ontology Request Broker (ORB). When using Phenex for data annotation, curators would invariably encounter the need to add new terms to the ontologies being used to most accurately characterize the anatomy descriptions presented to them. Using earlier versions of Phenex, data curators frequently had to switch tasks between data annotation and ontology development. That is, they paused data annotation, and changed software to a term tracker where they made a formal term request, or to an ontology editor where they added the desired term, deferring the formal term request. For the rich, interoperability-promoting, and community-developed ontologies used in Phenoscape, formal term requests often entail lengthy discussions. This process proved a major limitation to annotation efficiency, as a single paper could require dozens of individual requests. To address this, we decoupled data annotation from ontology development by creating an Ontology Request Broker (ORB) component in Phenex, building on the recently developed “Provisional Term” service provided by the National Center for Biomedical Ontology (NCBO) BioPortal as part of its application programming interface (API) [9]. The Phenex ORB component allows curators to submit requests for provisional terms directly from the curation interface and use them in data annotation. The requested terms can be reviewed asynchronously for inclusion in the appropriate ontology as official terms. Data files are later automatically updated to reference the appropriate permanent term ID.
2. Collaborative editing capability. Large-scale curation projects like Phenoscape typically employ multiple personnel that participate in data curation, prompting the need that curators be able to edit data files collaboratively, even if not necessarily concurrently. Phenoscape curators had previously been using, with mixed success, a software version control system (Subversion [10]) in order to ensure that each curator’s copy of the project files was up to date with respect to changes made by others and to avoid conflicting edits. However, many biologists are not accustomed to installing or using version control software, and its ease of use varies between operating systems. In contrast, file sharing services such as Dropbox [11] can satisfy collaborative editing requirements while being mostly transparent to the user, and they are already widely used by biologists. To mitigate the risk of curators inadvertently overwriting changes made to a file by another user, we added two basic features to Phenex: file change monitoring and continual autosave. Phenex now immediately notifies the user when the file they are currently editing changes and offers to reload it, and the continual autosave decreases the likelihood of a user having potentially conflicting yet unsaved edits.
3. Annotation consistency constraints. Phenotype descriptions often use the full expressivity of natural language, and as a consequence there are often several or even many ways to compose ontological annotations to represent the semantics of a description. If left unchecked, the resulting variability can hinder interoperability for automated integration and reasoning across annotations. To promote annotation consistency among curators, Phenoscape has, as have others, established curation guidelines [8]. Aside from consistency, the quality of annotations may also be affected by ontology terms applied in incorrect contexts, such as inapplicable taxonomic group, or inconsistently with its definition, such as using a quality term defined as inhering in a single entity to annotate a relationship between two entities and vice versa. To better report and identify problematic or missing annotations, Phenex now features a Consistency Review panel, which allows curators to obtain a growing variety of quality control reports, as well as an Annotation Checker panel, which provides the same reports for the currently selected annotation.

## Implementation

The version of Phenex described here has been archived at http://dx.doi.org/10.5281/zenodo.12370. Phenex is developed using the Java Swing graphical interface toolkit. It is built on the application framework developed for the OBO-Edit ontology editor [12], which provides the ontology object model, ontology reading capabilities, and configurable interface layout.

### Ontology Request Broker (ORB)

The Ontology Request Broker relies on three main components: a term request interface and web service client within Phenex, the Provisional Class web services provided by the NCBO BioPortal [13], and a standalone web user interface for updating requested provisional terms with permanent identifiers [14]. Before a user can issue provisional term requests, they must enter their BioPortal user ID and API key into a Phenex configuration panel. The Phenex term request client provides an entry panel that allows the user to request a provisional term with the given name and description, optionally with suggested superclass and synonyms. Phenex sends the request (via HTTP POST) to the Representational State Transfer (REST) [15] based BioPortal web service API, and receives in return a new unique URI to be used as a class identifier. Phenex adds this new term to the current ontology session, which makes it available for use in annotations. Annotations using provisional IDs are saved in output NeXML files in the same manner as any other term reference.

At application launch time, Phenex issues a HTTP GET request to the BioPortal API for all provisional terms submitted by the configured user. This allows Phenex to add all provisional terms previously requested by the user to the user’s ontology session for use in annotations. In addition, for any provisional terms in the BioPortal database that have since the initial request been associated with permanent IDs, Phenex automatically migrates any annotations using those terms to use the permanent ID instead. This is accomplished by marking those terms as ‘obsolete’ within the Phenex ontology session, and adding to their metadata a ‘replaced_by’ annotation with the permanent ID as its value. Phenex updates any loaded data by following ‘replaced_by’ relationships, whether these are due to “normal” ontology changes that are part of their regular maintenance, or due to provisional term resolutions. To enable collaborative usage of provisional terms, and to avoid duplicate requests within the project, Phenoscape curators use a shared BioPortal user ID.

Phenoscape curators assign permanent IDs to provisional terms using a separate ORB manager web interface [14], which is a simple client-side web application implemented using AngularJS [16] and the BioPortal web services API.

### Collaborative editing support

Phenex monitors the file from which the currently open document was loaded for changes. The implementation makes use of the ‘jpathwatch’ Java library [17]. Any changes to the file that are not due to Phenex saving the document are reported to the user, giving the user the opportunity to immediately reload the file, or to ignore the changes. If the user has unsaved changes, reloading the file would result in these changes being discarded. To minimize the likelihood of this situation, Phenex also provides a user-configurable option to autosave the file after any change is made. Phenex autosave is built on its previously existing undo–redo support.

### Consistency review

Annotation consistency rules are implemented by the AnnotationConsistencyChecker class within the Phenex source code. This class contains several rules focused tightly on the Entity–Quality model and the semantic implications of the structure of the Phenotype and Trait Ontology (PATO) [18]. For example, annotations must include both an entity and a quality term; PATO “relational qualities”, which are qualities that relate between two entities rather than inhering in a single one, must be provided with an additional “related entity”; and entities descending from Gene Ontology ‘biological process’ (GO:0008150) must be used only with descendants of ‘process quality’ (PATO:0001236). Phenex displays errors or warnings in two locations. The Annotation Checker panel displays any issues found for the currently edited phenotype annotation; this panel continuously updates its status as the user edits the annotation. The Consistency Review panel displays a list of errors for all annotations in the dataset; additionally it notes if there are incompletely annotated characters, which are characters for which some but not all states have annotations.

## Results and discussion

As a result of these new features in Phenex, Phenoscape curators using the tool report a smoother workflow, with fewer interruptions for tasks related to ontology maintenance or file synchronization.

**Figure 1:**
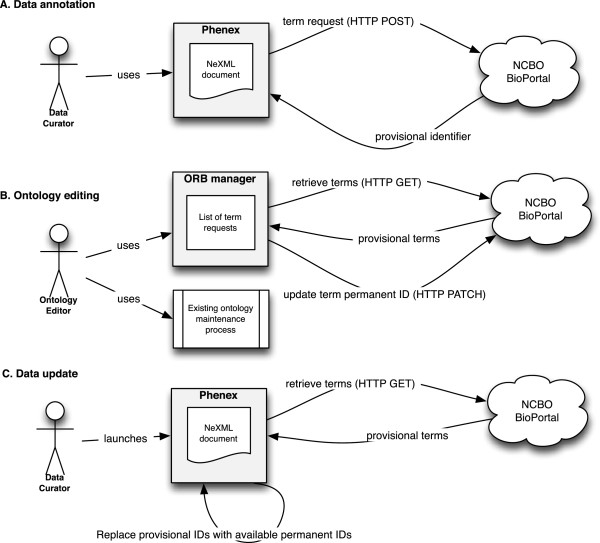
**Ontology term request workflow. A**. A data curator encounters a term which is not available from the relevant ontology. The curator composes and submits a term request. They receive a provisional term which can be used where needed. **B**. An ontology editor reviews the list of term requests using the Phenoscape ORB Manager web application. After creating an appropriate term in the ontology, the ORB Manager is used to update the permanent ID field in the provisional term record. **C**. When Phenex is launched, it checks for any provisional terms which have been assigned a permanent ID, and updates the current data file to reference the permanent IDs. These three phases of the workflow can occur repeatedly and largely independently of each other.

The most impactful of these new features is the Ontology Request Broker. Prior to its implementation, curators were required to manually keep track of unfinished annotations, while separately contributing missing terms to the relevant ontology. Submitting a new term to a community-developed ontology can be a complex process, possibly involving lengthy discussion with the expert community and ontology editors. The Ontology Request Broker in Phenex provides an annotation workflow that effectively decouples ontology editing from annotation work (Figure 1). When a missing term is encountered, the user can simply request it, without leaving Phenex, and receive a temporary identifier that they can immediately use within annotations. The term request consists of metadata such as the suggested label, superclass, definition, and possible synonyms. Later, without having to interrupt a data curation session, they can review their requested terms and manage the community vetting process for eventually adding those terms to the relevant ontologies.

Not every provisional term request necessarily results in an ontology term addition. In some cases, later review of the request reveals that a suitable term does exist already, but for some reason was not discovered. In this case the URI of the existing term can simply be entered as the permanent identifier for the provisional term. The failure to discover the already existing term may indicate that a naming variant is missing from the term’s synonym annotations, and the ontology editor may then choose to add the respective synonym.

When an annotation file that references provisional terms that have since been resolved is opened, Phenex automatically migrates the references from the respective provisional IDs to the corresponding permanent IDs. Phenex notifies the user in this case that annotations have been updated, and users do not need to take any other manual action to update their data with the correct term identifiers.

The workflow implemented in the Phenex ORB system has much in common with a detailed specification for an ontological term broker, with example implementation, created by the NeuroCommons project [19]. For our use case, we decided that enabling an asynchronous interaction between annotation and ontology development was the most critical aspect of the ORB, and we have not encountered a need for the more complex architecture described in the NeuroCommons system. For example, that system contains nine statuses that can be applied to requests, with associated transitions between them. We have found it sufficient to allow ontology curators to view the list of pending requests using our orb-manager web application, which is simply a lightweight interface for the BioPortal web services API. Ontology curators manage each term request and ontology update outside of the orb-manager application, which allows them to follow the procedures specific to each ontology. The ontology curator signifies completion of this procedure by adding a permanent ID to the term request.

By adopting the provisional term API provided by the NCBO BioPortal, we were able to avoid developing and hosting a request server back end. In practice, the functionality our system requires from the server is straightforward: generation of universally unique provisional IDs, storage of provisional request metadata, and the ability to update the request with a permanent ID. BioPortal, in the form of the WebProtégé application [20], does provide an interface for directly working with provisional term requests when editing an ontology, however none of the ontologies used by the Phenoscape project is managed via WebProtégé. For this reason, we have not attempted to more tightly integrate management of ontology editing into the ORB workflow.

As do many projects with significant semantic annotation goals, Phenoscape involves multiple personnel that participate in data curation. Even if curators do not annotate the same source concurrently, an effective curation workflow still requires that users can work collaboratively on the same source, and thus file. Phenex now allows simultaneous collaborative editing of annotation files through file or folder-sharing services (such as Dropbox). Phenex detects when a file changes on disk while it is being edited, and offers to reload it so the user works off of the most recent version, which avoids conflicting edits. To prevent unsaved edits from getting lost in this process, a user can configure Phenex to continually autosave a file after each edit. In our observations so far, the collaborative editing feature is frequently used by geographically separated users who are collaborating on data entry, for example by discussing the proper annotation of difficult phenotypes, and also by curators performing quality control of files produced by their peers. Even though the actual changes required to Phenex’s file handling were fairly minor, these features have in practice all but eliminated file conflicts, and have also facilitated training and collaborative work among Phenoscape curators. Rather than having to install software (such the originally used version control system) with which they are unfamiliar and which otherwise do not use, curators can now manage files in a familiar way, by simply saving to their local directory which synchronizes automatically to collaborators via the file sharing service used (such as Dropbox).

The annotation consistency constraints in Phenex augment a database-wide consistency review process that is already used by Phenoscape curators. Even though the currently implemented set of constraints is relatively small, curators find that having the quality control panels right in Phenex saves time and improves curator training by providing feedback about the most commonly encountered problems to curators as they perform their work. The initial utility of the feature suggests that a suitable direction for its further development could be to allow easier contribution of new quality control rules, for example by stating them declaratively rather than as Java code.

## Conclusions

We have added three new features to Phenex that allow users to focus more effectively on actual data annotation, while reducing the intrusion of ontology maintenance and file management into their workflow. The most significant of these features, both in effect and in engineering, is the Ontology Request Broker. Even though the introduction of required new terms to community ontologies is still a major component of the overall semantic annotation workflow, ontology editing and data editing can now be performed more independently and collaboratively.

## Availability and requirements

**Project name:** Phenex

**Project home page:**http://phenex.phenoscape.org/

**Operating system(s):** Platform independent

**Programming language:** Java

**Other requirements:** Java 1.7 or higher

**License:** MIT, http://opensource.org/licenses/MIT

**Any restrictions to use by non-academics:** none

## Abbreviations

API: Application programming interface
NCBO: National Center for Biomedical Ontology
ORB: Ontology Request Broker
PATO: Phenotype and Trait Ontology
REST: Representational State Transfer.
